# Soft drinks and 'desire to drink' in preschoolers

**DOI:** 10.1186/1479-5868-5-60

**Published:** 2008-12-02

**Authors:** Claire Sweetman, Jane Wardle, Lucy Cooke

**Affiliations:** 1 Department of Epidemiology and Public Health, Health Behaviour Research Centre, University College London, Gower Street, London, WC1E 6BT, UK

## Abstract

Interest in soft drink consumption has increased following a dramatic rise in intake over recent years. Research to date has focused primarily on general trends in consumption or on understanding the mechanism by which soft drink consumption may be linked to weight gain. It is clear however that there is considerable individual variability in the extent to which soft drinks are consumed and factors potentially influencing intake have received little attention. This study examines how the Child Eating Behaviour Questionnaire (CEBQ) construct 'Desire to Drink' (DD) relates to drink consumption, preferences and BMI-SDS.

Three hundred and forty six same-sex twin children (mean age 11.2 years; s.d. 0.54; 56% female; 53% dizygotic) were weighed, measured and reported their liking for milk, water, fruit juice, fruit squash and sweetened soft drinks. Mothers reported on their child's drink consumption and completed the CEBQ.

Scores on the CEBQ DD subscale were not significantly related to child BMI-SDS in this sample. Children scoring higher on DD had higher preferences for sugar-sweetened soft drinks (p = 0.016), fruit squash (p = 0.042) and milk (p = 0.020) than children scoring lower on the scale. DD was also positively related to more frequent consumption of sugar-sweetened soft drinks (p = 0.017) and low calorie soft drinks (p = 0.003). No relationship was observed between DD scores and liking for or intake of water or 100% fruit juice.

These findings suggest that the construct desire to drink in children is related to a liking for consuming sweetened drinks, and does not appear to simply denote greater thirst or hunger. This may have important implications for the ongoing development of dietary patterns and weight status in the longer term through an increased preference for sweet things in the mouth and a failure to compensate for calories provided by drinks.

## Background

Soft drink consumption has increased dramatically over recent years, with a five fold increase in the UK between 1974 and 1999 [1] and similar trends in the US. Soft drinks are often implicated as contributors to the rising rates of overweight and obesity, and although the relationship has not been unequivocally established, a recent systematic review concluded that there is a link between soft drink consumption and weight status in both adults and children [2]. A stronger, more consistent link was found between soft drink intake and increased energy consumption in a recent meta-analysis [3]. Both higher energy intake and higher weight may be due to poor compensation for the energy consumed in soft drinks [4]. As soft drinks have little or no nutritional value, they can be seen as 'empty calories', contributing to an overall increase in energy intake. The finding that soft drinks displace intake of milk [3,5] and increase preference for other high calorie foods [6] is also of concern, particularly for children in the process of developing dietary patterns and preferences.

Most of the literature on soft-drinks focuses on trends in consumption or common mechanisms relating consumption to excess energy intake, but there is also considerable individual variability in intake which has hitherto attracted little attention. The Child Eating Behaviour Questionnaire (CEBQ; [7]) includes a scale 'Desire to Drink' (DD), which measures differences in the quantity and frequency with which children want to drink.

To date, two studies have investigated the relationship between CEBQ-DD scores and adiposity in non-clinical samples. A study of 296 low-income African-American preschoolers [8] using two items from the DD scale found no relationship with weight, but Webber et al [9], using the full scale, found that DD scores were positively related to BMI in older children. However it is unclear whether DD relates to overall fluid consumption or to certain beverages in particular.

In order to take this work forward it is important to identify the behavioural correlates of 'desire to drink'. High scores denote a more frequent desire to have a drink, but give no indication of the *type *of drink. If DD is related to thirst, scores should be associated with consumption of all drinks, including water. If driven by hunger, a relationship with consumption of higher calorie, more satiating drinks (e.g. milk) would be predicted. Alternatively, if DD is related to a liking for sweet things in the mouth, high scorers would be expected to specifically consume higher quantities of sweetened beverages.

This study therefore examined the relationship between DD and liking and consumption of a range of beverages and adiposity in children aged 9–12 years.

## Method

### Participants

In 1997, 231 families of same-sex twins, drawn from the Twins Early Development Study (TEDS), were invited to take part in a study of children's eating habits (for further details of the TEDS sample see [10]). 214 families agreed, of whom 173 (81%) were followed-up seven years later when the children were aged on average 11.2 years. Data for these analyses come from the follow-up study.

### Data collection

Families were visited in their homes, where mothers and children were weighed and measured and completed measures of children's eating and activity habits and preferences.

#### Anthropometrics

Children's heights and weights were measured by trained researchers using a portable stadiometer and a digital Tanita scale. Adiposity was indexed with BMI- SDS relative to 1990 British norms [11].

#### Children's drink preferences

Children were presented with a list of drinks and were asked to indicate how much they liked each of them by ticking the appropriate box on a 5-point response scale ('I hate it', 'I don't like it', 'It's ok', 'I like it', 'I love it'). Liking for sweetened soft drinks (e.g. Coca Cola), fruit juice (100% pure), fruit squash (cordial), milk, and water was recorded.

#### Children's drink consumption

Drink intake was assessed using a food frequency questionnaire designed for British children [12] and completed by mothers. Responses were given using a 9-point response scale from 0–9 ('Never' to '7 days a week'). Frequency of consumption was recorded for sweetened soft drinks (e.g. Coca Cola), low-calorie soft drinks (e.g. Diet Coke), fruit squash, fruit juice (100% pure), and milk.

#### Children's desire for drinks

Mothers completed the CEBQ [7] for each child. This is a reliable and validated measure of eight dimensions of children's eating behaviour, with internal reliability coefficients ranging from 0.74 to 0.91 [7,13]. The CEBQ includes the Desire to Drink (DD) scale, composed of the following three items: 'My child is always asking for a drink'; 'If given the chance, my child would drink continuously throughout the day'; 'If given the chance, my child would always be having a drink'. Responses were scored from 1–5 ('never' to 'always') and DD scores were obtained by calculating the mean of the three item responses. Cronbach's alpha in the present sample was high (0.90).

### Statistical analysis

Analyses were performed using SPSS 14.0. Independent t-tests examined gender differences for all relevant variables. No significant gender differences were found, with the exception of frequency of consumption of unsweetened fruit juices, with boys consuming more (p < 0.001). Further analyses were therefore performed on the full sample in order to maximise statistical power. Pearson's product moment correlations were used to examine the relationship between DD scores and (i) BMI-SDS, (ii) liking for each of the five drink types, and (iii) frequency of consumption of each of the five drink types. Linear regression analyses allowed for adjustment for twin clustering, maternal education and zygosity.

## Results

### Participant characteristics

Participants were 346 same-sex twin children, with a mean age of 11.2 yrs (s.d. 0.54). BMI-SDS scores ranged from -2.42 to 3.54. 56% of the sample were female, 47% of twin pairs were monozygotic and 53% dizygotic. 68% of mothers completed their education by the age of 16 years, 19% by the age of 18 years and the remaining 13% completed university education.

### Desire to drink and BMI

DD scores were not significantly related to child BMI-SDS in this sample (p = 0.412).

### Desire to drink and preferences and frequency of drink consumption

Table 1 shows the results of unadjusted correlational and adjusted regression analyses of the relationship between DD scores, drink preference and frequency of consumption.

**Table 1:** Unadjusted correlational analyses and adjusted regression analyses for the relationship between scores on the DD subscale of the CEBQ, child drink preference ratings and intake

	Mean score (SD)	Association with DD score Unadjusted analyses		Association with DD score Adjusted analyses*		
		***r***	***p***	***B***	**CI (95%)**	***p(B)***

**Preferences for drinks**	(range 1–5)					
Milk	4.06 (1.18)	0.099	0.070	0.095	0.015–0.175	0.020
Sweetened soft drinks	4.22 (0.98)	0.144	0.008	0.120	0.023–0.217	0.016
Fruit squash	4.29 (0.89)	0.147	0.007	0.118	0.004–0.232	0.042
Unsweetened fruit juice	4.42 (0.84)	0.012	0.826	-0.005	-0.115–0.105	0.931
Water	4.14 (0.89)	0.005	0.929	0.015	-0.104–0.135	0.799
**Frequency of consumption**	(range 0–9)					
Milk	4.41 (1.20)	-0.19	0.725	0.027	-0.024–0.078	0.302
Sweetened soft drinks	2.71 (2.23)	0.222	0.000	0.071	0.013–0.129	0.017
Low calorie soft drinks	2.63 (2.74)	0.195	0.000	0.063	0.021–0.105	0.003
Fruit squash	7.21 (2.60)	0.121	0.027	0.037	-0.017–0.092	0.177
Unsweetened fruit juice	5.12 (3.18)	0.040	0.462	0.016	-0.025–0.057	0.433

* Analyses adjusted for twin clustering, maternal education and zygosity

A significant positive relationship was found between DD and preferences for milk, sweetened soft drinks and fruit squash, after controlling for twin clustering, maternal education and zygosity. There was no association between DD and preferences for fruit juice or water.

DD was also related to more frequent consumption of sugar-sweetened and low calorie soft drinks, but not to frequency of consumption of milk, fruit squash and fruit juice.

## Conclusion

Results from this study show that children with a greater desire to drink consume carbonated soft drinks more frequently than those with lower scores and have a stronger liking for sweetened soft drinks, both carbonated and still. While firm conclusions cannot be drawn from the current data, had it not been for the emergent association between DD and preference for milk in the adjusted analyses (albeit with an unchanged effect size from the unadjusted analyses), these findings are supportive of the hypothesis that 'desire to drink' in children may be related to a desire for sweet things in the mouth and is not simply a matter of thirst or hunger. Given evidence of an association between sweetened soft drink consumption and higher energy diets [3], the results suggest that DD warrants further investigation in an attempt to further clarify the behavioural correlates of the construct and any potential implications for future overweight and obesity.

Previous studies have shown that children are poor at compensating for calories consumed in drinks [4], so a greater desire to drink may be associated with increased total energy intake. Certainly our findings suggest that children with a stronger desire to drink may be likely to consume a significant amount of sugar, purely through beverage intake. Furthermore, preference for the taste of sweetened soft drinks is strongly related to intake [14] and has been linked to preferences for other high calorie and non-nutritious food and drink [6]. In this context, a desire for sweetened drinks may well be associated with less healthful dietary choices and increased risk of overweight and obesity in the longer term.

While we observed no relationship between DD scores and adiposity, in contrast to previous findings of Webber et al [9], this may have been the result of a lack of statistical power to detect what might be a small effect.

As this study is cross-sectional in nature it is not possible to determine causal relationships and it may be that more frequent consumption of sweetened beverages increases preference and desire to drink. Factors such as availability and accessibility are also likely to play a role, although these were not assessed in this sample.

As yet, little is known about the development and behavioural correlates of children's desire to drink, nor whether it can be modified and this is an area that warrants further scrutiny. In the meantime, parents should be encouraged to offer their children water when they ask for a drink. Even if initially rejected, previous research [15] would suggest that children will come to accept water after repeated exposure if alternatives, such as sweetened beverages, are not made available. It may be that such an intervention would reduce the desire to drink and is a worthwhile focus for future research.

## Competing interests

The authors declare that they have no competing interests.

## Authors' contributions

LC devised the study. JW contributed to the design of the study. LC and CS performed the statistical analyses. CS prepared the first full draft of the manuscript. LC and JW contributed to the writing of the manuscript.
